# Anti-Ro/SSA Antibodies and the Autoimmune Long-QT Syndrome

**DOI:** 10.3389/fmed.2021.730161

**Published:** 2021-09-06

**Authors:** Pietro Enea Lazzerini, Franco Laghi-Pasini, Mohamed Boutjdir, Pier Leopoldo Capecchi

**Affiliations:** ^1^Department of Medical Sciences, Surgery and Neurosciences, University of Siena, Siena, Italy; ^2^Veterans Affairs New York Harbor Healthcare System, State University of New York Downstate Medical Center, New York, NY, United States; ^3^New York University School of Medicine, New York, NY, United States

**Keywords:** anti-Ro/SSA antibodies, long QT syndrome, autoimmune cardiac channelopathies, hERG potassium channel, Torsades de Pointes, sudden cardiac death

## Abstract

Autoimmunity is increasingly recognized as a novel pathogenic mechanism for cardiac arrhythmias. Several arrhythmogenic autoantibodies have been identified, cross-reacting with different types of surface proteins critically involved in the cardiomyocyte electrophysiology, primarily ion channels (autoimmune cardiac channelopathies). Specifically, some of these autoantibodies can prolong the action potential duration leading to acquired long-QT syndrome (LQTS), a condition known to increase the risk of life-threatening ventricular arrhythmias, particularly Torsades de Pointes (TdP). The most investigated form of autoimmune LQTS is associated with the presence of circulating anti-Ro/SSA-antibodies, frequently found in patients with autoimmune diseases (AD), but also in a significant proportion of apparently healthy subjects of the general population. Accumulating evidence indicates that anti-Ro/SSA-antibodies can markedly delay the ventricular repolarization *via* a direct inhibitory cross-reaction with the extracellular pore region of the human-ether-a-go-go-related (hERG) potassium channel, resulting in a higher propensity for anti-Ro/SSA-positive subjects to develop LQTS and ventricular arrhythmias/TdP. Recent population data demonstrate that the risk of LQTS in subjects with circulating anti-Ro/SSA antibodies is significantly increased independent of a history of overt AD, intriguingly suggesting that these autoantibodies may silently contribute to a number of cases of ventricular arrhythmias and cardiac arrest in the general population. In this review, we highlight the current knowledge in this topic providing complementary basic, clinical and population health perspectives.

## Introduction

The long QT-syndrome (LQTS) is a cardiac electric disorder characterized by an abnormal prolongation of the heart rate-corrected QT interval (QTc) on the electrocardiogram (traditionally >440 ms; currently, >470 ms for men, and >480 ms for women) (1) which predisposes to life-threatening ventricular arrhythmias (VAs), specifically Torsades de Pointes (TdP) (1–3). TdP is a polymorphic ventricular tachycardia presenting with a typical pattern of twisting points that can rapidly degenerate into ventricular fibrillation (VF) and cause sudden cardiac death (SCD) (1). The more the QT prolongs the more the risk of TdP increases, it becoming high for QTc > 500 ms, very high for QTc > 600 ms (1). In addition, accumulating evidence indicates that when QT interval prolongs as the result of the specific lengthening of the terminal component of the T wave, from the peak to its end (Tpeak-Tend interval, Tp-Te), the risk to develop TdP is particularly important (4, 5).

The QTc on the electrocardiogram (ECG) is commonly used in the clinical practice as a proxy of the average action potential duration (APD) in ventricular cardiomyocytes, in turn determined by the sequential activation of several ion channels mediating inward depolarizing (sodium, Na^+^ and calcium, Ca^++^) or outward repolarizing (potassium, K^+^) currents, respectively (6). Whenever a dysfunction of one or more of these channels occurs leading to a net inward shift in the balance of currents (i.e., an increase of Na^+^ or Ca^++^ currents and/or a decrease of K^+^ currents), APD prolongs and, therefore, the QTc (6, 7). A wide number of etiologic factors can be responsible for LQTS, classically categorized as congenital, due to mutations in genes encoding for K^+^, Na^+^, or Ca^++^ channels and related regulatory proteins, or acquired (8, 9). While inherited forms are relatively rare, with an estimated prevalence of ~1:2,000 of apparently healthy newborns (8, 10), acquired LQTS is a quite frequent finding (11, 12), more commonly due to medications blocking the human *ether-à-go-go* related gene K^+^ channel (hERG-K^+^) carrying the rapidly activating component of the delayed outward-rectifying current (I_Kr_), or electrolyte imbalances (hypokalaemia, hypocalcaemia, hypomagnesemia) (1, 9). Other well-defined causes of acquired LQTS include structural heart diseases, bradyarrhythmias, liver and endocrine diseases, nervous system injuries, starvation, hypothermia, and toxins (9, 13). Although wide, this list of “conventional” risk factors is not able to account for all cases of LQTS/TdP occurrence (and recurrence) in the clinical practice (4) and for this reason in the recent years, intensive investigations were undertaken to identify previously unrecognized risk factors. As a result, an increasing number of novel, “non-conventional” QT-prolonging risk factors for acquired LQTS have been recently recognized, including human immunodeficiency virus infection (14), male hypogonadism (15, 16), heart failure with preserved ejection fraction (17), QT-prolonging foods (18), inflammation and autoimmunity (19, 20).

Regarding the autoimmune LQTS, the most investigated form is associated to the presence of circulating anti-Ro/SSA-antibodies, responsible for one of the first identified arrhythmogenic autoantibody-induced channelopathies (*autoimmune cardiac channelopathies*) (19, 21, 22). In fact, accumulating evidence exists that anti-Ro/SSA-antibodies exert significant electrophysiological effects on the heart *via* an inhibitory cross-reaction with the extracellular pore region of the hERG-K^+^ channel (23–27), leading to a higher propensity of developing LQTS (28–31) and VAs/TdP (24, 32, 33) in anti-Ro/SSA-antibody positive adults and newborns subjects. In this review, we highlight the current knowledge on this autoimmune associated LQTS form providing complementary basic, clinical and population health perspectives.

## Anti-Ro/SSA-Antibodies

Anti-Ro/SSA-antibodies, comprising the anti-Ro/SSA-52kD and anti-Ro/SSA-60kD sub-specificities, result from an autoimmune response against the two subunits of the intracellular ribonucleoprotein Ro (Ro52-kD and Ro60-kD) (34). They are polyclonal antibodies, usually of the IgG class, commonly found in patients with autoimmune diseases (AD) and beyond (34–36). In particular, anti-Ro/SSA-positivity is frequent in connective tissue diseases (CTD), primarily Sjögren's syndrome and systemic lupus erythermatosus (SLE) (34). In these disorders, anti-Ro/SSA-60kD sub-specificity has a more established direct pathogenic role than anti-Ro-52kD in the development of classical autoimmune manifestations (37, 38), also being associated with a higher prevalence of extraglandular features, especially vasculitis, and greater systemic activity (39, 40). Indeed, large studies have demonstrated that anti-Ro/SSA-antibodies can be also detected in a significant proportion of subjects of the general population (0.5-2.7%) (41–43), who are in most cases (60%) asymptomatic for AD (43), particularly when anti-Ro/SSA-52kD positivity occurs alone (44).

Large evidence exists that the trans-placental passage of anti-Ro/SSA-antibodies from the mother to the fetus causes the autoimmune-congenital heart block (aCHB) (45), a paradigmatic form of passively acquired autoimmunity (46, 47). Although the pathogenesis of this disorder is complex and only in part elucidated, many clinical and experimental data have demonstrated that an inhibitory cross-reaction between anti-Ro/SSA-antibodies and the L- and T-type Ca^++^-channels in fetal conduction system cardiomyocytes plays a key mechanistic role (48–54).

In the clinical practice, several laboratory methods are available for anti-Ro/SSA-antibody detection, the more commonly used being immunoenzymatic tests (ELISA, FEIA) and line-blot immunoassay (LIA), all based on recombinant Ro antigens use as substrate (34, 36, 55, 56). However, increasing evidence indicates that immuno-Western blot (iWB), using the native Ro antigen, is the most sensitive technique to reveal anti-Ro/SSA-positivity in the general population (36) as well as arrhythmogenic autoantibodies in aCHB (57), more frequently being identified as the anti-Ro/SSA-52kD subtype (57).

## Anti-Ro/SSA-Associated Long-QT Syndrome

### Clinical Data

The first studies showing an association between anti-Ro/SSA-antibodies and LQTS were performed in children in the early 2000s (Table 1). Cimaz et al. (28) reported that newbors/infants without aCHB from anti-Ro/SSA-positive mothers had longer QTc than anti-Ro/SSA-negative controls. Moreover, the same authors demonstrated that such alteration normalized spontaneously during the first year of life together with the disappearance of maternally-acquired anti-Ro/SSA-antibodies, thereby pointing to a functional and reversible interference on ventricular repolarization (59). Later, four independent groups provided data further supporting this association (Table 1). Gordon et al. (58) demonstrated that QTc was significantly prolonged in children from anti-Ro/SSA-positive mothers when compared to those from anti-Ro/SSA-negative mothers, with a more marked prolongation in siblings of a child with aCHB. Then, Jaeggi et al. (60) reported that in a Canadian cohort 116 anti-Ro/SSA-positive newborns/infants without aCHB, transient QTc prolongation was rather frequent, it being present in 15% of cases (60). Consistent data were more recently obtained by Friedman et al. (62) who analyzed the ECGs of 45 infants without aCHB born from anti-Ro/SSA-positive mothers and found that QTc prolongation > 2 standard deviations above historical healthy controls was present in 11% of subjects. Moreover, AlTwajery et al. (61) demonstrated that among 41 children affected with SLE, anti-Ro/SSA-positivity was associated with a higher prevalence of ECG abnormalities, particularly QTc prolongation > 450 ms. Finally, three cases of marked QTc prolongation complicated with ventricular tachycardia/TdP in infants from anti-Ro/SSA-positive mothers with aCHB are reported (63–65).

**Table 1:** Clinical studies showing an association between anti-Ro/SSA antibodies and QTc/TdP.

**References**	**Study population**	**Anti-Ro/SSA+ (*n*)**	**Anti-Ro/SSA– (*n*)**	**Main results**
**Newborns/children**				
Cimaz et al. (28)	Newborns of CTD mothers	21	7	Mean QTc significantly longer in anti-Ro/SSA-positive subjects; QTc prolongation > 440 ms in 42% of cases (vs. 0% in controls)
Gordon et al. (58)	Children of CTD mothers	38	7	Mean QTc significantly longer in children of anti-Ro/SSA-positive mothers
Cimaz et al. (59)	Children of anti-Ro/SSA-positive mothers	21	-	Concomitant disappearance of QTc prolongation and acquired maternal antibodies at 1 year follow-up
Jaeggi et al. (60)	Newborns/children of anti-Ro/SSA-positive mothers	116	-	Transient QTc prolongation > 440 ms in 15% of cases
AlTwajery et al. (61)	Children with SLE	16	25	Anti-Ro/SSA-positive patients showed higher prevalence of ECG abnormalities, particularly QTc prolongation > 450 ms
Friedman et al. (62)	Newborns/children of anti-Ro/SSA-positive mothers	45	-	QTc prolongation > 2 SD above historical healthy controls in 11% of cases
Duke et al. (63)	Newborn of an anti-Ro/SSA-positive mother	1	-	QTc prolongation and ventricular tachycardia
Wang et al. (64)	Child of an anti-Ro/SSA-positive mother	1	-	QTc prolongation and TdP
Mizuno et al. (65)	Child of an anti-Ro/SSA-positive mother	1	-	QTc prolongation and TdP
**Adults**				
Lazzerini et al. (29)	CTD	31	26	Mean QTc significantly longer and prevalence of QTc prolongation > 440 ms significantly higher in anti-Ro/SSA-positive subjects (58 vs. 0%)
Lazzerini et al. (32)	CTD	26	20	Mean QTc significantly longer and prevalence of QTc prolongation > 440 ms significantly higher in anti-Ro/SSA-positive subjects (46 vs. 5%); QTc prolongation significantly associated with the presence of complex ventricular arrhythmias
Bourrè-Tessier et al. (30)	SLE (two studies)	57 113	93 165	5.1-12.6-times higher risk of QTc prolongation in anti-Ro/SSA positive vs. negative group. The risk of QTc prolongation directly correlated with anti-Ro/SSA concentration
Lazzerini et al. (66)	CTD	25	24	Mean QTc significantly longer and prevalence of QTc prolongation ≥ 460 ms significantly higher in anti-Ro/SSA-positive subjects (48 vs. 17%); significant correlation between anti-Ro/SSA-52kD concentration and QTc duration
Pisoni et al. (67)	AD	55	18	Anti-Ro/SSA positivity significantly more frequent among CTD patients with QTc prolongation ≥ 440 ms (all patients with QTc prolongation were anti-Ro/SSA positive, 20 vs. 0%)
Sham et al. (68)	SLE	47	53	Mean QTc significantly longer in anti-Ro/SSA-positive subjects
Nakamura et al. (33)	TdP	1	-	QTc prolongation and TdP in an anti-Ro/SSA-positive woman without AD
Lazzerini et al. (24)	TdP	25	-	High prevalence of anti-Ro/SSA-52kD in unselected TdP patients (60%)
Perez-Garcia et al. (69)	SLE	66	-	Anti-Ro/SSA and anti-Ro/SSA-52kD levels significantly higher in patients with QTc prolongation, and linearly correlated with QTc duration
Tufan et al. (70)	CTD	15	39	QTc max, Tp-e and Tp-e/QT ratio higher in anti-Ro/SSA-52kD-postive vs. negative CTD patients (and HC, *n* = 22);Tp-Te duration strongly correlated with anti-Ro/SSA-52kD titer
Mostafavi et al. (71)	SLE	150	-	Anti-Ro/SSA positivity significantly associated with QTc prolongation > 440 ms
Hu et al. (72)	SLE	299	-	Anti-Ro/SSA positivity identified as one of the most important independent variables associated with QTc prolongation > 450 ms
Lazzerini et al. (31)	US Veterans	612	6,727	QTc prolongation (>470 ms in males/>480 ms in females) in 10% of anti-Ro/SSA-positive vs. 6.2% of negative subjects (marked QTc prolongation, >500 ms, 3.1 vs. 1.0%). Anti-Ro/SSA positivity independently associated with a 2-times higher risk of marked QTc prolongation (>500 ms; OR 2.27, 95%CI 1.34-3.87)

*CTD, connective tissue disease; AD, autoimmune disease; SLE, systemic lupus erythematosus; SSc, systemic sclerosis; HC, healthy controls; QTc, corrected QT interval; TdP, torsades de pointes; Tp-e, interval from the peak to the end of the T wave; Tp-e/QT ratio, interval from the peak to the end of the T wave/QT interval ratio*.

In agreements with these findings, several studies demonstrated an increased prevalence of QTc prolongation and VAs in adults with circulating anti-Ro/SSA-antibodies (Table 1). Our group was the first to provide evidence that anti-Ro/SSA-positive adults with CTD frequently show QTc prolongation (>440 ms in ~45-60% of cases) (29, 32), persisting throughout the 24 h and correlating with the risk of complex VAs (32). Later on, Bourré-Tessier et al. (30) conducted two consecutive studies on a larger cohort of SLE patients where anti-Ro/SSA-positivity was found to be associated with a 5-12-times higher incidence of QTc prolongation, with a correlation with autoantibody levels. This latter finding was confirmed and refined by our group, by demonstrating that only the serum concentration of the anti-Ro/SSA-52kD subtype significantly and specifically associated with QTc duration (66).

After these seminal studies, many other authors provided clinical evidence supporting the existence of a relationship between anti-Ro/SSA-antibodies and QTc prolongation risk in adults (Table 1). Pisoni et al. (67) demonstrated that among 73 AD patients, the prevalence of QTc > 440 ms was significantly higher in anti-Ro/SSA-positive (20%) vs. –negative subjects (0%). Consistent results were obtained by four subsequent studies, all conducted in SLE patients. Sham et al. (68) reported that mean QTc was longer in SLE subjects with, rather than without circulating anti-Ro/SSA-antibodies, while Mostafavi et al. (71) and Perez-Garcia et al. (69) found that anti-Ro/SSA-antibodies were more commonly detectable and at a higher concentration when SLE patients with QTc prolongation were compared to those with a normal QTc. Moreover, in a study using machine learning in 299 patients with SLE, Hu et al. (72) identified anti-Ro/SSA positivity as one of the most important independent variables associated with QTc prolongation > 450 ms in these subjects. Regarding the specific role of the anti-Ro/SSA-52kD subtype, Tufan et al. (70) reported increased QTc maximum and Tp-Te values in anti-Ro/SSA-52kD-positive CTD patients in comparison to negative patients and healthy controls. In addition, Perez-Garcia et al. (69) and Tufan et al. (70) found that anti-Ro/SSA-52kD levels were significantly associated with QTc and Tp-Te duration in SLE and CTD patients, respectively.

Further studies provided evidence that anti-Ro/SSA-antibodies, regardless of the presence or absence of a clinically evident CTD/AD, are *per se* associated with LQTS/TdP (Table 1). This is a very important point, as it intriguingly suggests that these autoantibodies may represent a concealed risk factor possibly contributing to life-threatening VAs/SCD events in the general population (21). In fact, after the early case report by Nakamura et al. (33) of recurrent TdP episodes in an otherwise healthy anti-Ro/SSA-positive woman with circulating anti-Ro/SSA-antibodies, our group more in general demonstrated that circulating anti-Ro/SSA-antibodies are silently found in a significant proportion of unselected patients presenting with TdP. By analyzing a prospective cohort of 25 TdP subjects consecutively collected from the general population, we found the presence of anti-Ro/SSA-52kD-antibodies in over 50% of patients, in most cases without a history of AD (24). In agreement with what was observed in children with aCHB (57) and patients with CTD (66), also in this case iWB was demonstrated to be the most sensitive laboratory technique in revealing arrhythmogenic autoantibodies. Strong support for these data is provided by a very recent population study conducted in a large cohort of 7339 US Veterans, including 612 anti-Ro/SSA-positive (31). In these subjects, circulating anti-Ro/SSA-antibodies were independently associated with a ~2-times higher risk of marked QTc prolongation (>500 ms), regardless the presence or not of history of CTD. Moreover, stepwise multivariate logistic regression analysis demonstrated that anti-Ro/SSA positivity was one of the most important contributors to marked QTc prolongation, with a significant synergy with most of the concomitant traditional QT-prolonging risk factors, including antimalarials (31). In fact, accumulating evidence demonstrates that this class of drugs, commonly used for the treatment of CTD patients, can inhibit the hERG-K^+^-channel (73–75) and promote LQTS development (76). Nevertheless, by stratifying Veterans according the antimalarials use, it was demonstrated that even in the absence of these drugs subjects who were anti-Ro/SSA-positive showed a prevalence of QTc > 500 two-fold higher than in those who were anti-Ro/SSA-negative (31).

Besides the aforementioned studies, it should be noted how other authors reported that adult or pediatric anti-Ro/SSA-positive patients showed increased QTc duration and/or QTc prolongation prevalence with respect to negative controls. However, such differences approached but did not reach the statistical significance, most likely because of the undersized samples used. This is the case of four additional studies reporting slightly longer mean QTc (Gordon et al., *p* = 0.06; Motta et al., *p* = 0.06) (77, 78) or higher proportion of QTc prolongation (Nomura et al., *p* = 0.08; Bourré-Tessier et al., wide 95%CI) (79, 80) in the presence of circulating anti-Ro/SSA-antibodies.

While this large body of data provides robust evidence for a clinically significant association between anti-Ro/SSA-antibodies and LQTS risk, some studies involving children (81, 82) or adults (83–87) reported apparently conflicting results. Several factors may account for these discrepancies, also possibly contributing to the significant variability in anti-Ro/SSA-associated QTc prolongation frequencies even reported by positive association studies (~10-60%) (25, 66). Firstly, given that the QT-prolonging effects seems to be specifically due to the anti-Ro/SSA-52kD subtype, and in a concentration-dependent manner (23, 24, 27, 66, 69, 70), it is likely that patients in these cohorts did not present circulating levels of this autoantibody sufficient to produce measurable electrocardiographic changes. Indeed, among different CTDs, a wide variability exists in terms of anti-Ro/SSA-52kD concentrations (for example, in systemic sclerosis patients the antibody level is typically low) (85, 88, 89), and in most of the negative association studies specific subtype assessment was not executed. In addition, most of these studies were retrospective and utilized different cutoffs to define QTc prolongation, these factors also potentially contributing to inconsistencies. This is the case, for example, of the study by Teixeira et al. (83) in which the QTc was considered as prolonged when >500 ms. As a result, only 10 out of 317 SLE patients showed QTc prolongation, a sample size that is underpowered for any statistical comparison between anti-Ro/SSA-positive (4/111, 3.6%) and -negative subjects (83). Data from the recent population study on US Veterans provide new important details, which provide support to the above considerations (31). In fact, in this large cohort, where only the qualitative data of anti-Ro/SSA-positivity was considered (no information on antibody subtypes and related concentrations available) the overall prevalence of QTc prolongation > 470 ms (males)/480 ms (females) in anti-Ro/SSA-positive subjects was 10% (vs. 6.2% in anti-Ro/SSA-negative, *p* < 0.001) (31), a percentage underestimated since several individuals without/with low levels of anti-Ro/SSA-52kD subtype were certainly present among those labeled as anti-Ro/SSA-positive. Notably, 3.1% of the subjects with circulating anti-Ro/SSA-antibodies (vs. 1.1% in anti-Ro/SSA-negative) showed QTc prolongation > 500 ms, proportions in part similar to those found by Teixeira et al., (83) but in this case very different from a statistical point of view (*p* < 0.001) due to the adequate power of the sample size (31).

Finally, as discussed in more details in the following section “Experimental Data,” anti-Ro/SSA-antibodies can concomitantly inhibit multiple cardiac ion channels, resulting in conflicting effects on APD, thereby on QT interval duration on the surface ECG (21, 90). Such a multifaceted impact on cardiomyocyte electrophysiology, along with the inherent (genetic and acquired) variability in cardiac ion channels reserves among different individuals (91, 92), may also significantly contribute to the reported discrepancies among clinical studies (21, 90).

### Experimental Data

Accumulating data from experimental studies based on *in-vitro, ex-vivo*, and *in-vivo* models (Table 2) (23, 24, 26, 27, 33) demonstrated that the QT-prolonging effect of anti-Ro/SSA-antibodies, specifically the anti-Ro/SSA-52kD subtype, is due to a specific cross-reaction with the cardiac hERG-K^+^ channel leading to an inhibition of the related current, I_Kr_ (21). The direct electrophysiological nature of such an effect can well explain why circulating anti-Ro/SSA-antibodies are *per se* associated with an increased risk of QTc prolongation/TdP in the clinical setting, regardless of the presence or not of an overt AD (24, 31).

**Table 2:** Basic mechanisms of anti-Ro/SSA-associated LQTS: data from experimental studies.

**References**	**Effect on hERG-K^**+**^**	**Effect on IKr**	**Effect on APD**	**Effect on QT interval**
Nakamura et al. (33)	direct binding in HEK293-hERG cells incubated with purified IgGs from an anti-Ro/SSA-positive TdP patient	chronic inhibition in HEK293-hERG cells incubated with sera/purified IgGs from an anti-Ro/SSA-positive TdP patient	-	-
Yue et al. (23)	1. direct binding in HEK293-hERG cells incubated with purified IgGs from anti-Ro/SSA-positive CTD patients with LQTS 2. direct binding in HEK293-hERG cells and guinea-pig ventricular tissue incubated with anti-Ro/SSA-positive sera from Ro52kD-immunized guinea-pigs 3. cross-reactivity with a 31–amino acid peptide corresponding to the pore-forming region (segment S5-S6) incubated with sera from anti-Ro/SSA-positive CTD patients with LQTS	1. acute inhibition in HEK293-hERG cells and/or guinea-pig ventricular myocytes incubated with sera/purified IgGs/affinity-purified anti-Ro/SSA-52kD antibodies from anti-Ro/SSA-positive CTD patients with LQTS 2. acute inhibition in HEK293-hERG cells incubated with anti-Ro/SSA-positive sera from Ro52kD-immunized guinea-pigs	prolongation in guinea-pig ventricular myocytes incubated with purified IgGs from anti-Ro/SSA-positive CTD patients with LQTS	prolongation at the surface ECG in Ro52kD-immunized guinea-pigs
Lazzerini et al. (24)	1. direct binding in HEK293-hERG cells incubated with purified IgGs from anti-Ro/SSA-positive TdP patients 2. cross-reactivity with a 31–amino acid peptide corresponding to the pore-forming region (segment S5-S6) incubated with sera from anti-Ro/SSA-positive TdP patients	acute inhibition in HEK293-hERG cells incubated with purified IgGs from anti-Ro/SSA-positive TdP patients	-	-
Fabris et al. (26)	-	acute inhibition in HEK293-hERG cells and guinea-pig ventricular myocytes incubated with sera from guinea-pigs immunized with a 31–amino acid peptide corresponding to the hERG pore-forming region (E-pore peptide) and cross-reacting with sera from anti-Ro/SSA-positive CTD patients with LQTS	prolongation in guinea-pig ventricular myocytes incubated with sera from E-pore peptide-immunized guinea-pigs	prolongation at the surface ECG in E-pore peptide-immunized guinea-pigs
Szendrey et al. (27)	1. direct binding to the extracellular S5-pore linker in HEK293-hERG cells incubated with commercial anti-Ro/SSA-52kD antibodies 2. decreased expression with enhanced endocytic degradation in HEK293-hERG cells incubated with commercial anti-Ro/SSA-52kD antibodies	chronic inhibition in HEK293-hERG cells and neonatal rat ventricular myocytes incubated with sera from anti-Ro/SSA-52kD-positive CTD patients or commercial anti-Ro/SSA-52kD antibodies	prolongation in neonatal rat ventricular myocytes chronically incubated with commercial anti-Ro/SSA-52kD antibodies	-

*hERG-K^+^, human ether-à-go-go related gene potassium channel; HEK293-hERG, human embryonic kidney-293 cells stably expressing hERG-K^+^; I_Kr_, rapidly activating component of the delayed outward-rectifying current; APD, action potential duration; CTD, connective tissue disease; IgGs, immunoglobulins G; LQTS, long-QT syndrome; TdP, torsades de pointes; ECG, electrocardiogram*.

Specifically, our group demonstrated that incubation of human embryonic kidney-293 cells stably expressing the hERG-K^+^-channel (HEK293-hERG) or guinea-pig ventricular myocytes with serum, purified IgGs, or affinity-purified anti-Ro/SSA-52kD obtained from CTD patients with LQTS was associated with an acute (minutes), concentration-dependent and reversible I_Kr_ inhibition (23). Moreover, the development of high levels of circulating anti-Ro/SSA-52kD antibodies in guinea-pigs immunized with the Ro52 antigen was associated with an evident prolongation of the APD measured in ventricular myocytes, as well as of the QTc measured at the surface ECG (23). Furthermore, by combining WB and ELISA experiments, we also provided evidence that anti-Ro/SSA-antibodies can directly cross-react with the hERG-K^+^-channel, specifically with the S5-S6 segments of the extracellular loop of the pore region where a significant sequence homology with the Ro52 antigen was demonstrated (23). Consistently, the immunization of guinea-pigs with a 31-amino acid peptide corresponding to this region of the hERG-K^+^-channel resulted in high levels antibodies able to block I_Kr_, prolong APD and QTc, in the absence of any structural change at the pathology examination of the myocardium (26). In addition, a recent Canadian study provided further mechanistic insights into anti-Ro/SSA-associated QTc prolongation, explaining its long-lasting persistence as observed in the clinical setting (27). In fact, these authors demonstrated that prolonged incubation of HEK293-hERG cells with anti-Ro/SSA-52kD-positive sera from patients with rheumatic diseases significantly decreased I_Kr_ compared to cells treated with autoantibody-negative patients' sera (27). Moreover, they showed that anti-Ro/SSA-52kD antibodies chronically facilitated hERG endocytic degradation by targeting the extracellular S5-pore linker region of the channel, and that these changes were associated with persistent I_Kr_ reduction and APD prolongation in neonatal rat ventricular myocytes (27).

The same mechanisms are implicated in anti-Ro/SSA-positive subjects who develop TdP, despite the absence of a manifest AD (Table 2). The first evidence was provided by Nakamura et al. (33) who demonstrated that serum and purified IgGs from an otherwise healthy anti-Ro/SSA-positive woman presenting with marked QTc prolongation and recurring TdP, cross-reacted with the hERG-K^+^-channel and chronically blocked I_Kr_ in HEK293-hERG cells. Our group confirmed and refined these findings in a prospective cohort of 25 consecutive TdP patients, including 15 (60%) with circulating anti-Ro/SSA-52kD antibodies, in most cases (13/15, 87%) without a history of AD (24). Again, sera and IgGs from anti-Ro/SSA-52kD-positive subjects significantly reduced I_Kr_ in HEK293-hERG cells, but also in guinea-pig ventricular myocytes, and recognized the hERG-K^+^-channel by specifically interacting with the S5-S6 segment of the extracellular loop of the pore-forming region (24).

Altogether, these data robustly support the hypothesis that a direct hERG-K^+^-channel blockade is the molecular mechanism underlying QTc prolongation and TdP observed in anti-Ro/SSA-positive subjects. However, it should be noted that anti-Ro/SSA-antibodies can also cross-react with and block cardiac Ca^++^-channels (48, 50–52, 54), responsible for opposite effects on APD. This view is supported by a mathematical modeling study which demonstrated how a simultaneous anti-Ro/SSA-associated inhibition of I_CaL_ during the plateau phase partly counterbalances the APD prolonging effect due to I_Kr_ decrease (26). Based on this evidence, it is likely that the inherent ion channel reserve which characterize each single subject (92) may significantly influence the overall impact of anti-Ro/SSA-antibodies on the duration of the QTc on the surface ECG, thereby contributing to explain the inconsistencies among clinical studies on the association of anti-Ro/SSA-antibodies and QTc prolongation (25). However, given that I_Kr_ physiologically activates after the T wave peak on the ECG (6, 93), a specific evaluation of the Tp-Te might represent a more accurate method to assess in the clinical setting, the discrete impact of anti-Ro/SSA-antibodies on this current. This also in consideration of the particularly important prognostic role that Tp-Te prolongation seems to have in predicting TdP risk (4, 5). In agreement with such premises, Tufan et al. (70) demonstrated that in anti-Ro/SSA-52kD-positive CTD patients Tp-Te was significantly prolonged when compared to anti-Ro/SSA-52kD-negative patients and healthy controls, even in those in whom the whole duration of the QTc was normal.

## Conclusions

**Figure 1:**
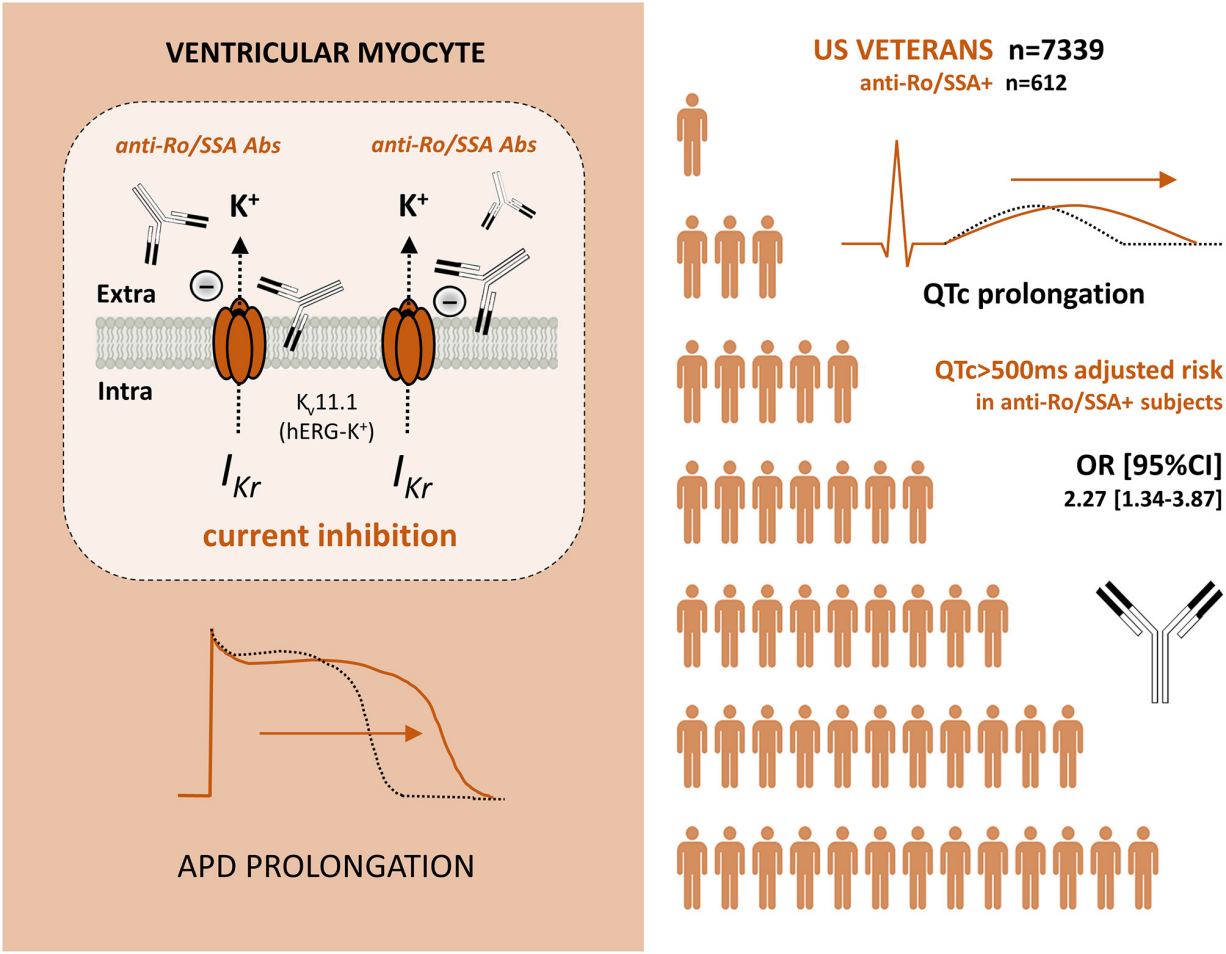
Anti-Ro/SSA antibodies, which can inhibit the I_Kr_ current by directly recognizing hERG potassium channel (brownish), are independently associated with an increased risk of marked QTc prolongation in a large cohort of Veterans (white). Abs, Antibodies; I_Kr_, rapidly activating component of the delayed outward-rectifying K^+^ current; hERG-K^+^, human *ether-à-go-go* related gene potassium channel; APD, Action potential duration; QTc, heart rate-corrected QT-interval; OR, odds ratio; CI, confidence interval.

Mounting evidence from clinical and experimental studies indicates that anti-Ro/SSA-antibodies can markedly affect the ventricular repolarization via a direct inhibitory cross-reaction with the extracellular pore region of the cardiac hERG-K^+^-channel, resulting in an increased predisposition to LQTS/TdP in anti-Ro/SSA-positive patients. Notably, recent data demonstrate that such a risk is increased independent of a history of overt AD, intriguingly suggesting that these autoantibodies may also silently contribute to a number of cases of VAs and cardiac arrest in the general population (Figure 1).

**Figure 2:**
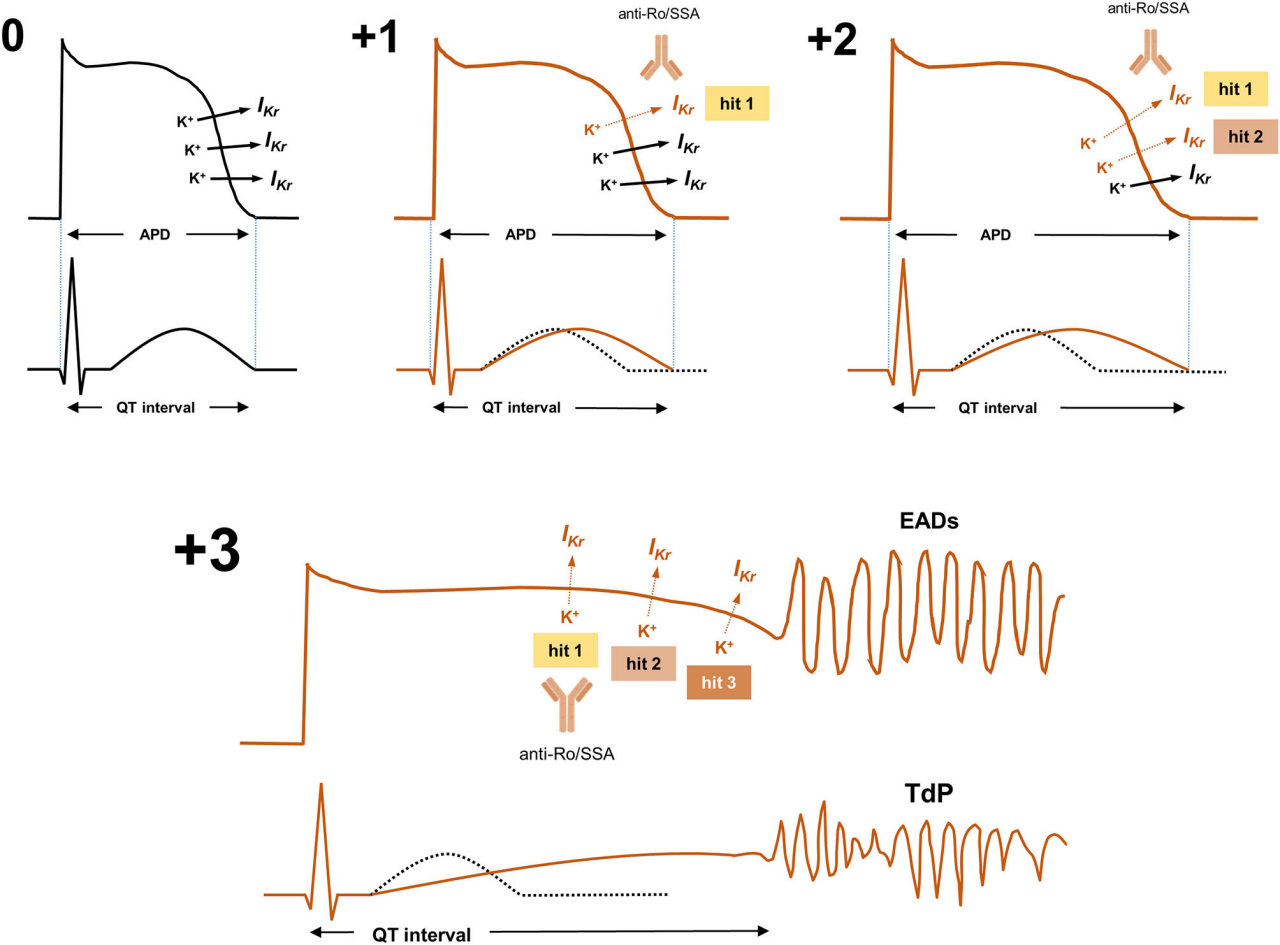
Anti-Ro/SSA-antibodies and the multi-hit theory of long-QT syndrome: given that manifold often-redundant ion channel mechanisms physiologically preserve ventricular APD, hence QTc length (repolarization reserve), many QT-prolonging risk factors (“hits”) need to be concomitantly present in a single patient to induce the marked disruption of ventricular repolarization necessary to the occurrence of life-threatening arrhythmias such as TdP. In the absence of QT-prolonging factors (0), I_Kr_ is preserved, and APD and QTc are normal. In subjects with the sole presence of arrhythmogenic anti-Ro/SSA antibodies partially inhibiting I_Kr_ (+1/hit 1), APD/QTc usually slightly/moderately prolongs (or even remains in the normal range, depending on pre-existing genetically-determined repolarization reserve). In these subjects, only the concomitant presence of other genetic or acquired QT-prolonging risk factors further inhibiting I_Kr_ and/or other key ion currents (such as drugs, electrolyte imbalances, etc.: +2/hit 2, +3/hit 3, etc.), can induce the marked APD/QTc prolongation critically required for TdP occurrence. I_Kr_, rapidly activating component of the delayed outward-rectifying K^+^ current; APD, Action potential duration; EADs, early afterdepolarizations; QTc, heart rate-corrected QT-interval; TdP, Torsades de Pointes.

In fact, although anti-Ro/SSA-antibodies alone cannot usually prolong QTc in a so critical manner to induce TdP development (similarly to all the other better recognized determinants of LQTS) (91, 94), nevertheless they can reduce the ventricular repolarisation reserve (92), thereby enhancing the arrhythmic risk when other conventional QT-prolonging factors (drugs, electrolyte imbalances, genetic mutations, etc.) are concomitantly present (*multi-hit theory*) (Figure 2) (24, 91, 95–99).

Based on these considerations and in some way referring to the existing guidelines on the approach to aCHB (100), it is recommended that anti-Ro/SSA-positive subjects receive serial ECGs and specific counseling about medications and management of other risk factors that may critically enhance the risk for QT-associated malignant arrhythmias. On the other hand, patients with “idiopathic” rhythm disturbances should be considered for specific anti-Ro/SSA testing (iWB technique is recommended for detecting arrhythmogenic anti-Ro/SSA subtypes), regardless the presence or not of a manifest AD, given that the demonstration of circulating antibodies may lead to innovative therapeutic opportunities. Indeed, in agreement with current recommendations for incomplete forms of aCHB (100) (and with several case reports showing the reversing effects of immunosuppressive therapy in anti-Ro/SSA–associated atrioventricular blocks in adults) (101–104), preliminary data from anti-Ro/SSA-positive CTD patients suggest that a short course immunomodulating treatment with corticosteroids is associated with a significant QTc shortening (104, 105). Larger studies are warranted to confirm these intriguing findings. Moreover, given that anti-Ro/SSA-antibodies prolong APD/QTc by directly reacting with a specific amino acid sequence of the hERG-K^+^ channel, a peptide-based therapy serving as a decoy to prevent autoantibody-channel binding may be another innovative approach, as preliminarily supported by *ex-vivo* data on sera from anti-Ro/SSA-positive TdP subjects (24).

## Author Contributions

PL: conception and design of the work and drafting the work. PL, FL-P, MB, and PC: final approval of the version to be published. FL-P, MB, and PC: revising the draft of the work critically for important intellectual content and agreement to be accountable for all aspects of the work in ensuring that questions related to the accuracy or integrity of any part of the work are appropriately investigated and resolved. All authors contributed to the article and approved the submitted version.

## Conflict of Interest

The authors declare that the research was conducted in the absence of any commercial or financial relationships that could be construed as a potential conflict of interest.

## Publisher's Note

All claims expressed in this article are solely those of the authors and do not necessarily represent those of their affiliated organizations, or those of the publisher, the editors and the reviewers. Any product that may be evaluated in this article, or claim that may be made by its manufacturer, is not guaranteed or endorsed by the publisher.
